# Fertility preservation: a case report of a newborn following 13 years of oocyte cryopreservation

**DOI:** 10.5935/1518-0557.20220070

**Published:** 2023

**Authors:** Ricardo Azambuja, Mariangela Badalotti, Lilian Okada, Luciana Segurado Cortes, Marta Ribeiro Hentschke, Alvaro Petracco

**Affiliations:** 1 Fertilitat - Reproductive Medicine Center, Porto Alegre, RS, Brazil

**Keywords:** cryopreservation, assisted reproductive technology, oocytes, fertility preservation, pregnancy

## Abstract

**Objective:**

Oocyte cryopreservation enables the storage of genetic material, especially in situations where the ovarian function is compromised, also for women desiring to postpone maternity. Before 2012, oocyte cryopreservation was still experimental, and the success of the procedure was uncertain; however, it was the only possibility that women had for fertility preservation. Thus, we aim to report a case of a birth after 13 years of elective oocyte cryopreservation.

**Case Description:**

At 49 years of age, the patient returned to our reproductive center with the desire to get pregnant, using oocytes that had been frozen for 13 years. The endometrium was prepared, and the oocytes were thawed using the slow procedure method. Four of the six oocytes thawed survived (66%) and were inseminated; three fertilized and started their development. The transfer of two embryos on the third day of development was performed. Clinical pregnancy was confirmed via ultrasound and came to term with the birth of a healthy boy.

**Discussion:**

Although the vitrification procedure has shown to be a better cryopreservation technique when compared to slow freezing, the latter represented an important role when patients wanted to cryopreserve oocytes in the early 2000s. Even many years later, this technique reveals its efficacy, preserving the viability and quality of oocytes stored in nitrogen tanks. After a literature review, this case seems to be the largest interval between oocyte cryopreservation and its use, with achieved pregnancy, in Brazil.

## INTRODUCTION

Social cryopreservation of oocytes has received increasing attention as a method of preserving reproductive potential. Currently, the largest group of women seeking to preserve fertility are those who wish to delay pregnancy for personal reasons. However, the cryopreservation of mature oocytes is not only an option for single ladies but also for those who may have partners but are concerned about the practical limitations of embryo cryopreservation.

The first successful pregnancies after oocyte cryopreservation using the slow-frozen method were reported by Chen (1986). However, concerns about the technique and the low success rates at that time ended up being a limitation of progress in this field of study.

In contrast to cryopreservation of embryos and sperm, oocyte cryopreservation is technically more challenging. We know that the oocyte is the largest cell in the mammalian organism, and since oocytes contain more water and thus are more sensitive to cryoinjury from ice crystal formation, its cryopreservation is notably difficult (Gosden, 2005). Some structures may be exposed from freezing techniques, such as the meiotic spindle, cytoskeleton, cortical granules, and zona pellucidae. However, it is known that by using special freezing techniques, nearly 70% of cryopreserved oocytes survive the freeze-thaw process, and probably around 90% survive with the newest protocols (Wang *et al*., 2013).

Until 1999, social (elective) freezing was performed using the slow cryopreservation technique. Even knowing the limitations of this technique, some women chose this method thinking about postponing motherhood. Kuleshova *et al*. (1999) published the first successful oocyte vitrification, changing the perspectives for this field of study, and in 2013, the American Society for Reproductive Medicine (ASRM) and the Society of Assisted Reproductive (SART) stopped considering oocyte preservation as experimental. Currently, the use of oocyte cryopreservation increased 25% from 2015 to 2016 (SART, 2021), and it is possible to say that fertilization and pregnancy rates are similar to in vitro fertilization (IVF) of fresh oocytes (ASRM, 2013).

However, what happened to patients who had tried to preserve fertility in the past using slow freezing is somewhat unclear. There is a lack of data in the literature, making it difficult to perform statistics on the success rates of thawing, fertilization, and pregnancy rates, using this technique that was so important in the past.

Thus, this paper aimed to report a case of an IVF experience using cryopreserved oocytes from a 13-year period, including survival, fertilization, cleavage, pregnancy rates, and the outcome of the pregnancy that resulted in a live birth.

## CASE DESCRIPTION

This is a case report study conducted at a reproductive medicine center in southern Brazil that included a couple who wanted pregnancy, and she had frozen oocyte as elective fertility preservation 13 years ago. The study was approved by the Ethics Committee of the Pontifical Catholic University of Rio Grande do Sul (Number: 3.663.607).

Before the vitrification technique became routine in Assisted Reproduction laboratories, a few clinics around the world offered the possibility of oocyte cryopreservation through the slow procedure technique. From 2000 to 2008, our Center, in partnership with Saint Barnabas Clinic (USA), offered this technique to patients that wished to cryopreserve their oocytes for many different reasons (Azambuja *et al*., 2002).

Thus, in 2008, a 36 years-old patient was referred to our clinic for oocyte cryopreservation to delay maternity. Following ovarian stimulation with recombinant follicle-stimulating hormone (FSH) and human menopause gonadotrophin (hMG) within the protocol with Gonadotropin-Releasing Hormone (GnRH) agonist, nine oocytes were retrieved, and six were mature (metaphase II). The oocytes were cryopreserved in a choline-based solution with a cryoprotector and frozen using the slow cryopreservation method (Stachecki *et al.*, 1998). In 2021, the patient, at 49 years old, returned to the clinic with the desire to thaw her oocytes and get pregnant. She had oligomenorrhea and a normal uterus seen in the ultrasound. Her partner was 66 years old, and his semen had 0.1 ml of volume, concentration equal to 90x10^6^ sperm/ml, and 20% motility. The endometrium was prepared with estradiol valerate 6 mg/day orally. When it reached 9 mm of thickness, vaginal progesterone 600 mg/day was started. At this moment, the oocytes were thawed using the slow procedure method. Four of the six oocytes thawed survived (66%) and were inseminated by intracytoplasmic sperm injection (ICSI); three fertilized and started their development. The transfer of two embryos happened on the third day of development: one embryo was starting compaction, and the other had six cells with 15% fragmentation, suitable for the day of development. The third embryo stopped its development following six days in culture. Twelve days after the transfer, βeta-hCG was 64 mIU/dL, three days later it was 215 mIU/dL, and on the 18^th^ day, it reached 2.415 mIU/dl. Fifteen days later, the ultrasound showed a gestational sac with one embryo with fetal heartbeats, compatible with six weeks of pregnancy. Three weeks later, another ultrasound was compatible with nine weeks of gestation. The pregnancy went to term, and the patient delivered a healthy male neonate at 38 weeks of pregnancy, with 3360g of birthweight and 50 cm birth length, and received Apgar score of nine at the 1^st^ and 5^th^ minute.

## DISCUSSION

The present study aimed to report a case of a live birth after 13 years of slow oocyte cryopreservation. To the best of our knowledge, this is the longest period of frozen oocyte for elective fertility preservation in Brazil, with delivery documented. However, there are few studies that reported related cases worldwide.

Quintans *et al*. (2002) published the first pregnancies and normal births using oocytes that were cryopreserved in a choline-based medium. In this study, 12 patients (21-41 years old) had oocytes cryopreserved in a modified phosphate-buffered saline medium, in which sodium chloride was replaced by choline chloride. A slow-freezing, rapid-thawing protocol was used, and oocytes were inseminated by ICSI. Median oocyte survival and fertilization rates were 63% and 59%, respectively. The overall implantation rate was 25%. Six clinical pregnancies were achieved; two of these pregnancies went to term resulting in the birth of two babies (Quintans *et al.,* 2002).

In 2008, a group from Bologna, Italy, reported a live birth after the transfer of a single blastocyst derived from a human oocyte cryopreserved for 5 years. A 39-year-old woman with tubal infertility and her partner with male-related infertility. Oocyte cryopreservation (in 1.5 mol/L 1,2-propanediol and 0.3 mol/L sucrose by slow freezing- rapid thawing protocol) happened when the patient was 34 years old. The oocyte was thawing after 5 years of cryostorage. Four of six mature frozen oocytes survived after thawing. Three of them were injected by ICSI and fertilized, and one developed into a five-cell embryo on day 3. This embryo developed into a blastocyst and was transferred on day 6. A healthy female neonate weighing 3,410 g was born (Parmegiani *et al*., 2008).

Quintans *et al*. (2012) from Buenos Aires, Argentina, reported a live birth after 11 years and 7 and a half months of cryopreserved human oocytes resulting in a live birth. At that time, the case represented the longest storage period. They reported a case of a 45-year-old woman who received embryos from IVF by ICSI with her own oocytes that were cryopreserved (slow freezing in a low-sodium medium) when she was 33 years old. From seven metaphase-II oocytes thawed, five survived, four were fertilized, and two cleaving embryos were transferred on day 3. A diamniotic dichorionic term pregnancy was achieved, ending with the delivery of two healthy girls. Two years later, the same research group (Urquiza *et al*., 2014) reported a case of a 38-year-old woman who received embryos from IVF by ICSI with her own oocytes that were cryopreserved by slow freezing in a low-sodium medium for 14 years and 6 months before, when she was 24 years old. From six metaphase-II oocytes thawed, two survived, one was fertilized, and a cleaving embryo was transferred on day 3. A single-term pregnancy was achieved, ending with the delivery of a healthy girl.

Oocyte survival is either all or none, which is in stark contrast to embryo freezing, where as much as 50% or more of the cells survive, the embryos can be considered for transfer. Oocyte survival is dependent on the method and experience of the embryologist. Tucker *et al.* (1998) suggested that since the cell needs to survive 100%, it is unlikely to have over 70% survival rate for frozen-thawed oocytes for the slow freezing technique. Boldt *et al.* (2006) used a choline-based media for freezing-thawing oocytes, reported a survival rate of 59.5%. Azambuja *et al.* (2005b) reported a 63% survival rate; however, only 8 oocytes were thawed. This study found similar results, with 4 out of 6 oocytes surviving cryopreservation. Stachecki, in 1998, using the same choline-based medium as the presented case reported, stated higher survival rates with donor oocytes, reaching 90% at times; however, media manufacture and laboratory conditions, not to mention oocyte quality, can all be considered as factors that could affect outcomes (Stachecki, 1998). Indeed, the true effectiveness of any medium and procedure is the result obtained in one’s clinic, and therefore may have different reports in the literature.

Boldt *et al*. (2003) reported 59% fertilization in frozen-thawed oocytes. Also, Quintans *et al*. (2002) inseminated 58 oocytes after thawing, of which 33 (56.9%) were fertilized. These reports, which also used different choline-based freezing medium and protocols, obtained similar fertilization rates, although somewhat lower (Boldt *et al*., 2003; Quintans *et al*., 2002). The 75% fertilization rate found in this case report is lower than the 100% and 80% reported by Azambuja *et al*. (2002; 2005b), but quite similar to the 69.2% reported by Azambuja *et al*. (2005a). The results from this study are lower than the 84% obtained by Grifo & Noyes (2010), even though a conventional sodium-based freezing medium was used.

The cleavage rate reported was 100%, like previous reports by Azambuja *et al.* (2005a,b) and Porcu *et al*. (2000), where both reported 100% cleavage. Petracco *et al.* (2006) also reported a high cleavage rate (91.8%) and found no difference in embryo quality when embryos from frozen-thawed oocytes were compared to fresh fertilized oocytes (Petracco *et al*., 2006).

The birth weight found in our report is higher than the 3271g reported by Ludwig & Diedrich (2002), in which the newborns developed from fresh oocytes undergoing IVF or ICSI, with no cryopreservation involved. The gestational age found in our report is similar to the 38.6 weeks reported by Ludwig & Diedrich (2002), in 301 children born by ICSI.

Regarding malformation, Porcu *et al*. (2000) did not find any malformation in 13 children born from frozen oocytes, similar to the results reported by Boldt *et al.* (2006), Quintans *et al.* (2002), and Grifo & Noyes (2010) with 6, 2, and 13 children, respectively; similar observations were reported by Azambuja *et al.* (2005a,b).

Concern has been expressed historically about the use of oocyte cryopreservation, particularly the potential for damage to the metaphase spindle, causing the risk of chromosomal anomalies (Pickering *et al.*, 1990). The results of several recent studies suggest that the risk of spindle damage is low, and while the spindle is lost during freezing, the spindle will normally reassemble after thawing and culture (Stachecki *et al*., 2004; Coticchio *et al*., 2005). Noyes *et al.* (2009), in an extensive review of 936 oocyte cryopreservation births (532 from slow-frozen oocytes and 392 from vitrified oocytes), reported only 12 congenital anomalies. In this report, they also described that the incidence of birth anomalies found in babies born from cryopreserved oocytes does not appear different from that of infants born through natural conception. The presented case report agrees, as there was no incidence of any malformation found or related to any of the 42 babies born (Azambuja *et al*., 2011). Therefore, we have been able to use existing technology to provide our patients with a reasonable chance of success.

Although currently, the vitrification procedure has shown to be a better cryopreservation technique when compared to slow-frozen oocytes, the latter represented an important role when patients wanted to cryopreserve oocytes in the early 2000. Even many years later, this technique has shown its efficacy, preserving the viability and quality of oocytes stored in nitrogen tanks, allowing oocyte utilization with success.

It is important to highlight that oocyte cryopreservation offers more flexibility for the female partner compared to frozen embryos. However, centers are proposing a combination of fertilizing some oocytes, creating embryos for cryopreservation, and cryopreserving some mature oocytes to provide women with future flexibility.

It is also important to point out that the cryopreservation process, even embryo or oocytes, does not guarantee the preservation of fertility. Even if there is a good embryo or gametes survival, this is not a guarantee of pregnancy, specially depending on the women’s age at frozen eggs.

After literature review, this case seems to be the longest interval between oocyte cryopreservation and its use with a birth in Brazil.

## References

[r1] ASRM (2013). Practice Committees of the American Society for Reproductive
Medicine and the Society for Assisted Reproductive Technology. Mature oocyte
cryopreservation: a guideline. Fertil Steril.

[r2] Azambuja R, Stachecki J, Badalotti M, Michelon Petracco A. (2002). Nascimento de feto a termo após congelamento de
oócitos. JBRA Assist Reprod.

[r3] Azambuja RM, Badalotti M, Okada L, Michelon J, Badalotti F, Petracco A. (2005a). Results of oocyte cryopreservation using choline-based freezing
medium. Fertil Steril.

[r4] Azambuja R, Badalotti M, Teloken C, Michelon J, Petracco A. (2005b). Successful birth after injection of frozen human oocytes with
frozen epididymal spermatozoa. Reprod Biomed Online.

[r5] Azambuja R, Petracco A, Okada L, Michelon J, Badalotti F, Badalotti M. (2011). Experience of freezing human oocytes using sodium-depleted
media. Reprod Biomed Online.

[r6] Boldt J, Cline D, McLaughlin D. (2003). Human oocyte cryopreservation as an adjunct to IVF- embryo
transfer cycles. Hum Reprod.

[r7] Boldt J, Tidswell N, Sayers A, Kilani R, Cline D. (2006). Human oocyte cryopreservation: 5-year experience with a
sodium-depleted slow freezing method. Reprod Biomed Online.

[r8] Chen C. (1986). Pregnancy after human oocyte cryopreservation. Lancet.

[r9] Coticchio G, Bonu MA, Bianchi V, Flamigni C, Borini A. (2005). Criteria to assess human oocyte quality after
cryopreservation. Reprod Biomed Online.

[r10] Gosden RG. (2005). Prospects for oocyte banking and in vitro
maturation. J Natl Cancer Inst Monogr.

[r11] Grifo JA, Noyes N. (2010). Delivery rate using cryopreserved oocytes is comparable to
conventional in vitro fertilization using fresh oocytes: potential fertility
preservation for female cancer patients. Fertil Steril.

[r12] Kuleshova L, Gianaroli L, Magali C, Ferraretti A, Trounson A. (1999). Birth following vitrification of a small number of human oocytes:
case report. Hum Reprod.

[r13] Ludwig M, Diedrich K. (2002). Follow-up of children born after assisted reproductive
technologies. Reprod Biomed Online.

[r14] Noyes N, Porcu E, Borini A. (2009). Over 900 oocyte cryopreservation babies born with no apparent
increase in congenital anomalies. Reprod Biomed Online.

[r15] Parmegiani L, Fabbri R, Cognigni GE, Bernardi S, Pocognoli P, Filicori M. (2008). Blastocyst formation, pregnancy, and birth derived from human
oocytes cryopreserved for 5 years. Fertil Steril.

[r16] Petracco A, Azambuja R, Okada L, Michelon J, Oliani A, Badalotti M. (2006). Comparison of embryo quality between sibling embryos originating
from frozen or fresh oocytes. Reprod Biomed Online.

[r17] Porcu E, Fabbri R, Seracchioli R, De Cesare R, Giunchi S, Caracciolo D. (2000). Obstetric, perinatal outcome and follow up of children conceived
from cryopreserved oocytes. Fertil Steril.

[r18] Quintans CJ, Donaldson MJ, Bertolino MV, Pasqualini RS. (2002). Birth of two babies using oocytes that were cryopreserved in a
choline-based freezing medium. Hum Reprod.

[r19] Quintans CJ, Donaldson MJ, Urquiza MF, Carretero I, Pasqualini RA, Horton M, Pasqualini RS. (2012). Live birth of twins after IVF of oocytes that were cryopreserved
almost 12 years before. Reprod Biomed Online.

[r20] SART - Society of Assisted Reproductive Technology (2021). Birmingham.

[r21] Stachecki JJ, Cohen J, Willadsen SM. (1998). Cryopreservation of unfertilized mouse oocytes: the effect of
replacing sodium with choline in the freezing medium. Cryobiology.

[r22] Stachecki JJ, Munné S, Cohen J. (2004). Spindle organization after cryopreservation of mouse, human, and
bovine oocytes. Reprod Biomed Online.

[r23] Tucker MJ, Morton PC, Wright G, Sweitzer CL, Massey JB. (1998). Clinical application of human egg
cryopreservation. Hum Reprod.

[r24] Urquiza MF, Carretero I, Cano Carabajal PR, Pasqualini RA, Felici MM, Pasqualini RS, Quintans CJ. (2014). Successful live birth from oocytes after more than 14 years of
cryopreservation. J Assist Reprod Genet.

[r25] Wang CT, Liang L, Witz C, Williams D, Griffith J, Skorupski J, Haddad G, Gill J, Wang W. (2013). Optimized protocol for cryopreservation of human eggs improves
developmental competence and implantation of resulting
embryos. J Ovarian Res.

